# Tumor Microenvironment Based on Extracellular Matrix Hydrogels for On-Chip Drug Screening

**DOI:** 10.3390/bios14090429

**Published:** 2024-09-05

**Authors:** Xiaoyan Liu, Jinxiong Cheng, Yingcan Zhao

**Affiliations:** 1 Institute for Health Innovation & Technology, National University of Singapore, Singapore 117599, Singapore; 2 Department of Biomedical Engineering, Southern University of Science and Technology, No. 1088 Xueyuan Road, Nanshan District, Shenzhen 518055, China; 3 Department of Bioengineering, Swanson School of Engineering, University of Pittsburgh, 3700 O’Hara Street, Pittsburgh, PA 15260, USA; jic259@pitt.edu; 4 Environment Science Programme, Department of Life Sciences, Beijing Normal University-Hong Kong Baptist University United International College, Zhuhai 519087, China

**Keywords:** microfluidics, dECM hydrogels, drug screening, nanomedicine, tumor microenvironment

## Abstract

Recent advances in three-dimensional (3D) culturing and nanotechnology offer promising pathways to overcome the limitations of drug screening, particularly for tumors like neuroblastoma. In this study, we develop a high-throughput microfluidic chip that integrates a concentration gradient generator (CGG) with a 3D co-culture system, constructing the vascularized microenvironment in tumors by co-culturing neuroblastoma (SY5Y cell line) and human brain microvascular endothelial cells (HBMVECs) within a decellularized extracellular matrix (dECM) hydrogels. The automated platform enhances the simulation of the tumor microenvironment and allows for the precise control of the concentrations of nanomedicines, which is crucial for evaluating therapeutic efficacy. The findings demonstrate that the high-throughput platform can significantly accelerate drug discovery. It efficiently screens and analyzes drug interactions in a biologically relevant setting, potentially revolutionizing the drug screening process.

## 1. Introduction

Recent advances in cancer research highlight the urgent need for more effective and precise drug screening models, particularly given the significant threat posed by cancers such as neuroblastoma, one of the most challenging malignancies to treat [1,2,3]. Two-dimensional (2D) cell cultures often fail to replicate the complex structures and dynamics of the microenvironments in vivo, leading to less predictive and realistic drug responses [4,5]. This limitation has shifted focus towards co-culture systems with 3D that better mimic the physiological context of tumor cells [6,7]. A common example of 3D culture is tumor spheroids, which provide a robust model for facilitating complex cellular communications within a construct, more closely simulating the microenvironmental conditions of tumors in vivo [8]. Additionally, dECM hydrogels, collected from porcine fat, exhibit excellent biocompatibility and biodegradability [9]. These hydrogels can simulate the tumor microenvironment, promoting cell growth and maintaining the 3D structures of a solid tumor [10,11]. Three-dimensional culture based on dECM hydrogel in high-throughput drug screening, especially for anticancer drugs, has become increasingly important [12]. The 3D models provide enhanced studies of cellular morphology, the enrichment of cancer stem cells, and the evaluation of therapeutic efficacy [13,14,15,16].

Functional nanoparticles are crucial in cell proliferation, migration, wound healing, tumor growth, and neural differentiation [17,18]. However, understanding cellular responses to varying concentrations of nanomedicines is essential [19]. A labor-intensive manual process and the need for advanced platforms hinder the study of the ideal concentration and high-throughput drug screening. Microfluidic-based gradient concentration generators, which precisely control the microenvironment and enable real-time monitoring, offer significant advantages in tumor research by allowing for parallel processing in high-throughput drug screening [20,21,22,23,24,25]. These systems have advantages, including low-volume and high-throughput characteristics [26]. However, due to particle aggregation and uneven distribution, generating uniform nanoparticle gradients within microfluidic systems remains a challenge [27]. This highlights the need for microfluidic platforms that can handle nanoparticle dilution as effectively as those for small molecules and proteins. Developing the platforms would streamline the drug development process, making it faster and more cost-effective while maintaining high-throughput capabilities. In addition, traditional drug screening systems are insufficient to accurately simulate the complex tumor microenvironment in vivo. This limitation affects the clinical accuracy of drug screening. By combining a gradient concentration system with a 3D chip that mimics the tumor microenvironment, the integrated platform allows for the precise control of drug concentrations and the dynamic monitoring of cancer cell responses within the tumor microenvironment, thereby enhancing the throughput and accuracy of drug screening and facilitating the identification of more effective drugs for tumors.

**Figure 1:**
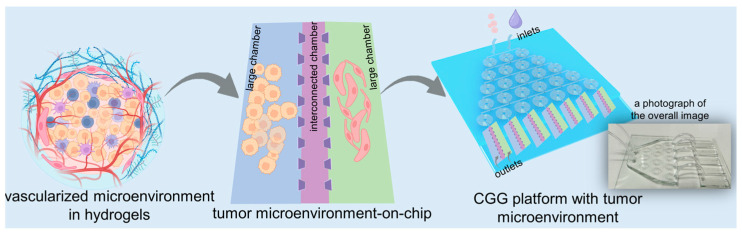
Schematic diagram of a high-throughput platform consisting of an automated CGG chip with a 3D vascularized microenvironment in tumors based on the dECM hydrogels. A photograph showing the overall image of the 3D tumor chip connected to the CGG chip as a proof of concept.

In this study, we develop a high-throughput microfluidic chip capable of automated gradient dilution for nanomedicines. This high-throughput platform integrates an automated CGG chip with a 3D tumor chip for co-culturing SY5Y cells and HBMVECs within dECM hydrogels (Figure 1). The 3D tumor chip is connected to the CGG chip by polyethene tubes. The polystyrene tube has an inner diameter of 0.020 inches, an outer diameter of 0.060 inches, and a length of 1 inch. A photograph displays the overall image of the 3D tumor chip connected to the CGG chip with five outlets, serving as a proof of concept (Figure 1). The neuroblastoma with vascular microenvironment encapsulated in hydrogels benefits from this precise control over drug concentration in the CGG module, enabling targeted studies of drug interactions within a biologically relevant setting. By combining robust 3D dECM culture environments with precise gradient control, the platform facilitates a high-throughput screening system that enhances the ability to simulate an in vivo microenvironment and accurately assess the therapeutic potential and mechanisms of nanomedicines, thereby paving the way for rapid and efficient drug discovery processes.

## 2. Materials and Methods

The design of the CGG chip and 3D microfluidic chip: We designed a CGG chip consisting of spiral channels with the width of 200 μm and the depth of 50 μm according to the previous study [28]. The 3D microfluidic chip is designed, consisting of two larger chambers and a smaller chambers in between. The larger chambers have a width of 4 mm, and the smaller chambers have a width of 2 mm; both have a length of 8 mm. The chambers are separated by micro barriers in between.

The fabrication of the CGG chip and the 3D microfluidic chip: We fabricated these microfluidic chips using poly-(dimethylsiloxane) (PDMS) (Sylgard 184; Dow Corning, Midland, MI, USA) by soft photolithography. The PDMS solution was mixed with a curing agent in a 10:1 mass ratio to form the PDMS pre-polymer. This pre-polymer was then poured onto an SU-8 master mold on a silicon wafer. After degassing in a vacuum chamber for 20 min and baking the pre-polymer at 80 °C for 60 min, the PDMS block was peeled off the mold, and we punched holes at the inlets and outlets. The patterned PDMS was then sterilized under UV light overnight. The two modules were bonded with glass and connected with tubes for further use.

The preparation of dECM hydrogel: dECM hydrogel was first obtained from DAT. DAT was collected from porcine fat. Porcine fat was obtained from the commercial market. The decellularization of porcine fat was conducted in the following steps: porcine fat was stored at a temperature of −20 °C for preservation; it was thawed at 37 °C in a water bath and then cut into small pieces ~1 × 1 × 1 cm^3^. After stratification, the middle layer and the lower layer were removed. The tissues were washed in PBS repeatedly to remove blood on the surface before the next step of agitation in different solutions. Tissues were agitated in water for 30 min at 37 °C, and then in 1 M NaCl for 2 h at 37 °C, and this process was repeated in water for 30 min at 37 °C. The tissues were washed three times in PBS solution, frozen at −80 °C for 24 h, and thawed in a 37 °C water bath. This freeze–thaw cycle was repeated for 3–5 cycles. The tissues obtained after the cycle were ground by a homogenizer at 12,000 rpm for 5 min and centrifuged at 1200× *g* for 5 min afterwards. The supernatant was discarded, and the precipitation was collected, while the fat in the middle layer was ground and centrifuged repeatedly until fat remained. Eventually, all precipitation was collected and agitated in 1% Triton-X 100 (MilliporeSigma, Darmstadt, Germany) at room temperature for 1 h and washed with distilled water three times for 30 min every time. To further remove the fat, the precipitation was immersed in isopropanol overnight at 37 °C. After the removal of isopropanol, the precipitation was immersed in 1M NaCl with 100 μg/mL DNase and 100 μg/mL RNase for agitation overnight at 37 °C, and centrifuged at 1000× *g*, 4 °C for 3 min. The precipitation obtained was washed three times with distilled water for 30 min every time.

After decellularization, DAT was stored at −80 °C for 24 h and lyophilized with the temperature set as −50 °C and vacuum degree <20 Pa for 24 h. The lyophilized DAT was pulverized and solubilized by pepsin treatment (1 mg/mL pepsin and 10 mg/mL DAT in 0.01 M HCl) under stirring for 48–72 h at 4 °C; the solubilization is carried out under sterilized conditions. It was stored at 4 °C for further use. The dECM hydrogel was obtained by adjusting the pH value of the pre-gel solution to 7.4 with the addition of 0.1 M NaOH and 10× PBS. The volume ratio of the pre-gel DAT solution, NaOH and 10× PBS are 10:1:1.1 in this experiment. To prevent gelation, the adjustment of pH is conducted on ice.

Cell culture: HBMVECs were cultured in DMEM medium (Gibco, Grand Island, NY, USA) with 10% FBS and 1% penicillin/streptomycin (P/S). SY5Y cells were maintained in F12/DMEM medium (Gibco, Grand Island, NY, USA) with 10% FBS and 1% P/S. These cell lines were cultured in cell incubators containing 0.5% CO_2_ at 37 °C. Cells were stained with Vybrant^®^ DiO (Invitrogen, Carlsbad, CA, USA) to monitor the migration of cells. Cell viability was evaluated by a Toxicity Assay Kit (Solarbio, Beijing, China).

Construction of 3D tumor microenvironment in dECM hydrogel: Cells were mixed with a pre-gel solution to achieve a density of 1 × 10^6^ mL^−1^ at each chamber. The HBMVEC and SY5Y cells within dECM hydrogels were slowly injected into the 3D tumor chip through the outlets. To mimic the tumor microenvironment, SY5Y cells and HBMVECs were cultured in each chamber, respectively. After the introduction, the cells were cultured in the 3D tumor chip for 12 h before being connected to the CGG chip. The nanoparticles were intermittently injected into the 3D chip for 7 days to minimize potential impacts of flow on the cells.

The calculation of the gap closure: The extent of gap closure in the migration assay is quantified by defining it based on the edges of the individual cells.

Statistical analysis: All experiments in the study were repeated at least 3 times for each condition. Statistical analysis of the experimental data was conducted with student *t*-test. There was statistical significance when *p* < 0.05.

## 3. Results

### 3.1. Spiral Microfluidic Chip for Generating the Gradient Concentration

**Figure 2:**
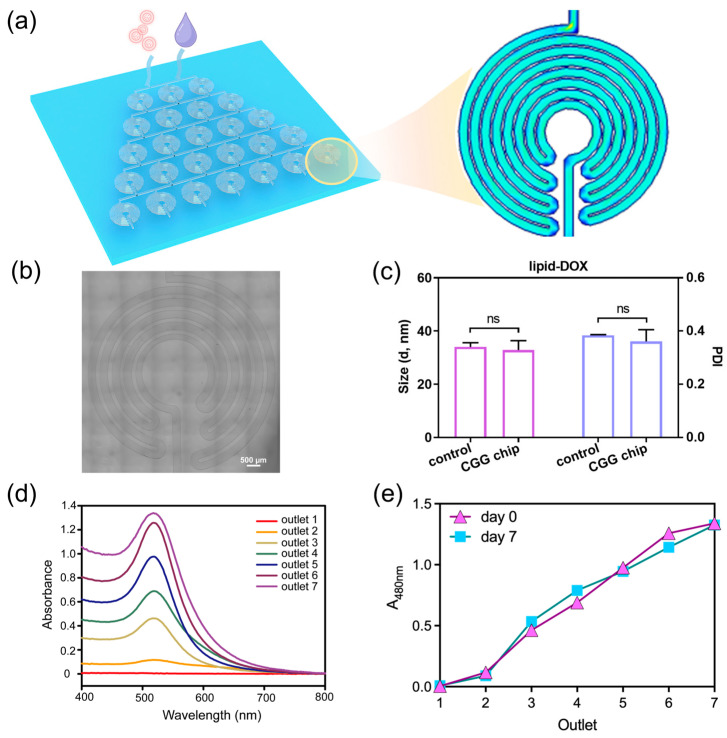
Characterization and performance of the CGG chip for drug dilution. (**a**) Schematic of the CGG chip for generating gradient concentrations of nanoparticles, the pink particles represent lipid-DOX nanoparticles, and the purple droplet represents the culture medium; the blue area represents low flow velocity, the green area represents medium flow velocity, and the red area represents high flow velocity in the velocity simulation. (**b**) image of the unit of CGG chip. (**c**) Hydrodynamic size and PDI of the lipid-DOX nanoparticle before/after flowing through the CGG chip, “ns” indicates that the *p*-value shows no significant difference; (**d**) the absorption spectra of lipid-DOX nanoparticles with different concentrations; (**e**) the absorbance of lipid-DOX nanoparticles at 480 nm flowing from different outlets on day 0 and day 7.

To address the challenges of nanoparticle dilution, we develop a microfluidic chip as an automated dilution module (Figure 2a). We design the CGG featuring compact disk-shaped mixers with an increasing number of diluted units. Each unit consists of turns and symmetrically arranged curved channels, where the fluid generates Dean flow to enhance the mixing of the nanoparticle solution (Figure 2b). This design not only increases the number of turns within the mixer but also significantly shortens the mixing distance, thereby ensuring uniform dilution without aggregation. The optimal conditions can be identified by adjusting the flow rate, which generates a linear concentration gradient.

The CGG chip can split fluid streams into equivalent sub-streams at each stage of the mixing units. These streams are continuously combined and mixed to produce a linear concentration gradient at the output, effectively preventing nanoparticle aggregation. We explore the capabilities of the CGG chip for preserving the mono-dispersion of lipid nanoparticles. The result shows that there was no significant difference in the hydrodynamic diameter and polydispersity index (PDI) of the lipid nanoparticles before and after flowing through the chip (Figure 2c). It indicates that the lipid nanoparticles can maintain their original size and dispersion after passing through the chip, demonstrating the excellent performance of the CGG chip in preserving the dispersion of the lipid nanoparticles.

To further investigate the capabilities of the CGG chip for generating gradient concentrations, we use lipid-doxorubicin (lipid-DOX) nanoparticles to quantify the gradient concentration generation at the flow rate of 1 μL/min to 10 μL/min. Since DOX has a characteristic absorption peak at 480 nm, we test the UV–vis spectra of the lipid-DOX nanoparticles from different outlets. The absorption spectra show that the intensity of the absorption peaks at the seven outlets gradually increase at the flow rate of 10 μL/min (Figure 2d). By quantifying the absorbance at 480 nm, we find that the CGG chip can generate a linear gradient concentration at the flow rate of 10 μL/min (Figure 2e). To evaluate the stability of the gradient concentration of nanoparticles generated by the CGG chip over time, we test the nanoparticle concentration downstream of the CGG chip on day 0 and day 7. The results show that a linear concentration gradient was still maintained on day 7, indicating that the nanoparticle concentration remained stable during this period (Figure 2e). These results demonstrate the precision of the CGG chip in generating gradient concentrations. The CGG platform significantly simplifies the screening process for generating gradient nanoparticle concentrations for anti-tumor studies, thereby reducing experimental burden while enhancing accuracy. This module enables the rapid identification of optimal concentrations, accelerating the discovery and validation of new nanomedicines.

### 3.2. Construction of Human Neuroblastoma Tumor with Vascular Microenvironment on Chip

**Figure 3:**
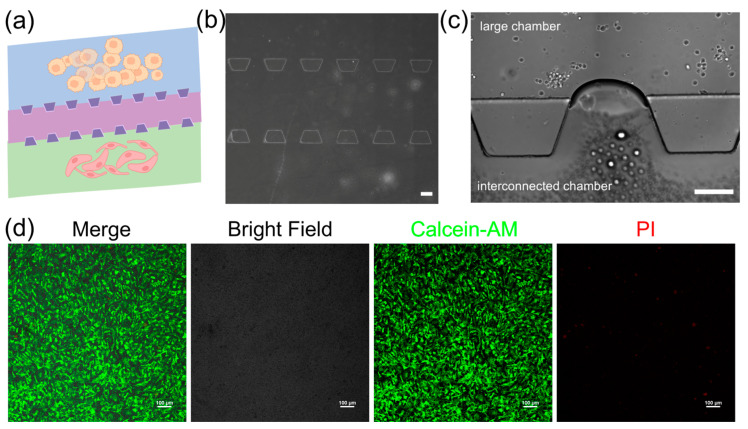
The iPSC-derived neuronal organoids in the hydrogels. (**a**) Schematic of the neuroblastoma tumor with vascular microenvironment on the 3D microfluidic chip, scale bar, 200 μm; yellow: SY5Y cells, pink: HBMVE cells (**b**) microscopic image of the 3D microfluidic chip, scale bar, 200 μm; (**c**) image of cell culture in the 3D microfluidic chip; (**d**) viability of cells loaded with dECM hydrogels in the 3D microfluidic chip, scale bar, 100 μm.

The 3D microfluidic chip in this study is designed to mimic the vascular microenvironment in neuroblastoma tumors by coculturing SY5Y cells and HBMVE cells (Figure 3a). The chip consists of two large cell culture chambers and one interconnected chamber. These chambers are separated by micro barriers, which are established through microchannels that allow for cell migration (Figure 3b). The connected design ensures effective nanoparticle introduction and uniform distribution across all sections via a network of channels, providing a dynamic controlled environment essential for examining drug efficacy and cellular response in the dECM hydrogels. The dECM hydrogels are used to culture neuroblastoma cells and assess their ability to support their growth (Figure 3c). We characterize the morphology of cells embedded inside dECM hydrogels using confocal laser scanning microscopy (CLSM). The images show that cells spread with high cell viability in the dECM hydrogels, indicating they have adapted well to 3D culture (Figure 3d). It shows that dECM hydrogels are biocompatible and supportive and can replicate a physiologically relevant tumor microenvironment. The tumor chip based on the dECM hydrogels can facilitate cellular functions that are necessary to study cell behavior and screen drugs.

**Figure 4:**
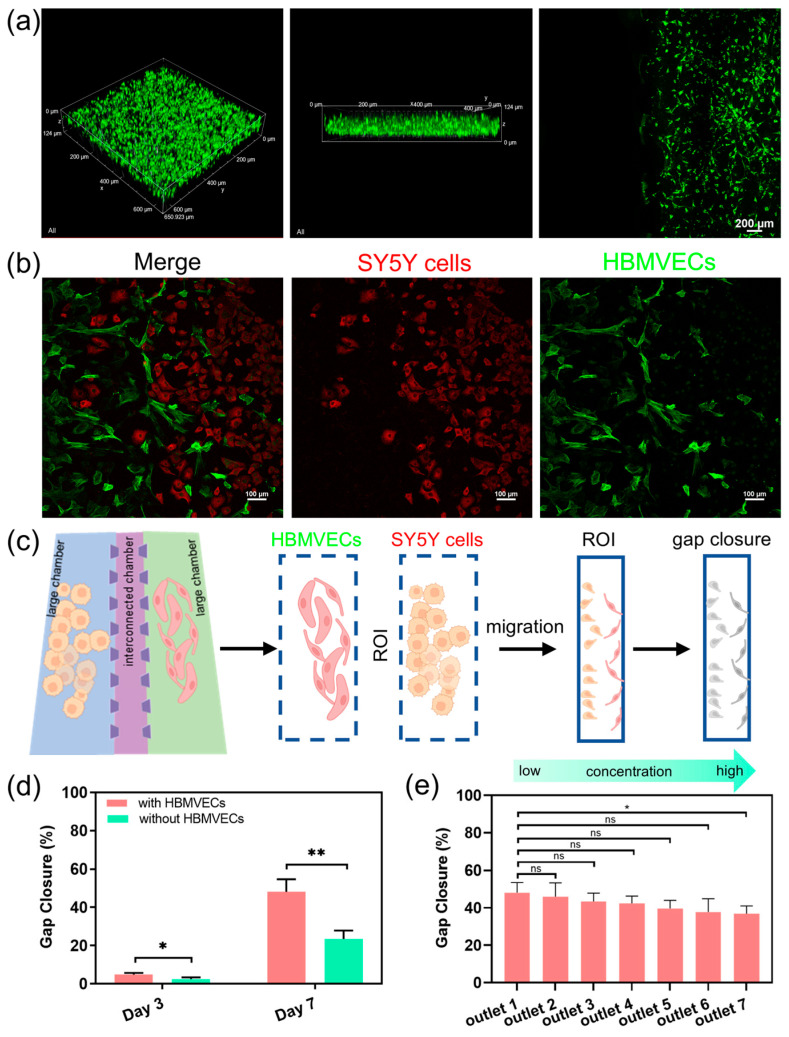
Evaluation of the neuroblastoma tumor with vascular microenvironment on-chip and drug screening; (**a**) fluorescence images of the cancer cells maintained in the dECM hydrogels in the cell culture chamber; (**b**) cell coculture and cell migration of the SY5Y cells and the HBMVECs in the microfluidic chip; (**c**) illustration of the measurement method of the gap closure of the microchannels. (**d**) Evaluation of the migratory of cancer cells with/without HBMVECs at different time points; (**e**) the change in the percentage of gap closure in the region of interest with the treatment of gradient concentrations of the lipid-DOX nanoparticles. Statistical significance was calculated using student *t*-tests, * indicates *p* < 0.05, ** indicates *p* < 0.01, and ns represents non-significant differences (*p* ≥ 0.05).

We further use the dECM hydrogels to construct a human neuroblastoma microenvironment, with which we can investigate more complex interactions between cells and drug responses. We evaluate the distribution of cancer cells within the cell culture chamber following their migration through the dECM hydrogels. The images show that the cells not only maintained their 3D morphology but also displayed active migration between chambers through the microchannels (Figure 4a). The vascular endothelial cells form networks resembling capillaries, whereas cancer cells interact closely, simulating the tumor–vascular interactions. The images show significant cellular integration and migration, indicating a strong microenvironment for studying tumor dynamics. The interactions between neuroblastoma cells and vascular endothelial cells show that the chip can replicate complex cell–cell interactions within a vascular microenvironment in neuroblastoma tumor (Figure 4b). We also quantify the capabilities of the cell migratory by calculating the gap closure of the microchannels. The microchannel regions are defined as the region of interest (ROI), and the gap closure is defined by the edge of the individual cells to quantify the extent of gap closure in the migration assay (Figure 4c). The results show that the migratory ability of cancer cells is significantly higher in the presence of HBMVECs compared to the absence of HBMVECs at different time points (Figure 4d). This observation highlights the application of the CGG chip for diluting nanomedicines, thereby investigating the bioeffects of nanomedicines on cells. We investigate the effects of lipid-DOX nanoparticles on tumor metastasis and angiogenesis over a period of 7 days. The quantitative results indicate that after treatment with gradient concentrations of lipid-DOX for 7 days, the metastatic potential of the cancer cells decreased (Figure 4e). This finding demonstrates a dose–response relationship between tumor metastasis and the concentration of lipid-DOX nanoparticles. The integrated concentration gradient generator and tumor microenvironment platform exhibit dual functions of automated gradient concentration generation and tumor vascular microenvironment simulation. This platform provides a high-throughput system for cancer therapy, offering crucial insights into the interactions between drugs and the tumor microenvironment.

## 4. Discussion

In this study, we successfully developed a high-throughput, automated microfluidic platform. This platform not only uses the dECM hydrogels as a substrate to simulate the vascular microenvironment in tumors, but also integrates a gradient concentration generator for the precise control of lipid-DOX nanoparticles concentration, increasing the throughput of drug screening on the chip. The automated platform can handle multiple channels, each conducting separate experiments under different conditions, showcasing its high-throughput capability. Additionally, the capability of the platform to rapidly screen a range of concentrations in a short amount of time further highlights its efficiency. The tumor chip can dynamically study how neuroblastoma interacts with the vascular microenvironment. By using dECM hydrogel, we can maintain an environment for mimicking the natural tissue of neuroblastoma, closely simulating physiological tumor conditions. The dECM hydrogel, with its structure and composition similar to the natural extracellular matrix, provides a 3D biological environment, allowing tumor cells to grow and interact in a way that is closer to in vivo conditions. In addition, the integration of the gradient concentration generator is another highlight of this platform. It can generate linear concentrations of lipid-DOX nanoparticles on the chip, which is crucial for drug screening and optimizing drug concentration. By precisely controlling drug concentration, we can evaluate the effects of different doses on cancer cells with the vascular microenvironment, thereby increasing the efficiency and accuracy of drug screening. The automated platform can accelerate the process of nanomedicine development and holds great potential in advancing drug evaluation based on the tumor microenvironment. For example, it can be used to study the response of cancer cells to nanomedicines within different tumor microenvironments and to monitor the dynamics of cancer cells treated with nanomedicines at varying concentrations. Overall, the automated platform provides a powerful tool for cancer research and drug screening. It can facilitate the study of tumor microenvironments and the development of nanodrugs with broad application prospects.

## Data Availability

All data required to assess the conclusions of the study are included within the paper. Any additional inquiries can be addressed to the corresponding authors.
